# Impact of signal strength on quantitative retinal and choriocapillaris flow measurement from optical coherence tomography angiography

**DOI:** 10.1038/s41598-022-08781-1

**Published:** 2022-03-18

**Authors:** Jae Jung Lee, Ji Eun Lee, Srinivas R. Sadda, Sung Who Park, Iksoo Byon

**Affiliations:** 1 Department of Ophthalmology, Pusan National University School of Medicine, Yangsan, South Korea; 2 Biomedical Research Institute, Pusan National University Hospital, Busan, South Korea; 3 Lee Eye Clinic, Busan, South Korea; 4 Doheny Eye Institute, Pasadena, CA USA; 5 Department of Ophthalmology, David Geffen School of Medicine at UCLA, Los Angeles, CA USA; 6 Department of Ophthalmology, Pusan National University Hospital, 179 Gudeok-ro, Seo-gu, Busan, 49241 South Korea

**Keywords:** Optical techniques, Eye diseases

## Abstract

We evaluated the impact of signal strength (SS) on quantitative measurements from optical coherence tomography (OCTA). Twenty healthy-volunteers were included. A neutral density filter (NDF) was attached to spectral-domain OCTA (SD-OCTA) and swept-source OCTA (SS-OCTA). All subjects were imaged with both devices three times using three different conditions: no filter, NDF0.3, and NDF0.6. For SD-OCTA, SS decreased from 10.0 to 8.2 and 4.0 with the NDF0.3 and 0.6, respectively. The vessel density (VD) and vessel length density (VLD) of the superficial capillary plexus (SCP) decreased when SS decreased from 10 to 8.2, but no further decrease when SS changed from 8.2 to 4.0. The flow metrics of the deep capillary plexus (DCP) did not change. For SS-OCTA, SS decreased from 10 to 9.5 and 7.2. The VD and VLD of the SCP and DCP decreased when SS decreased, except for the VD of the DCP when SS changed from 10 to 9.5. The choriocapillaris flow deficits significantly increased along with the decrease in SS. Quantitative flow parameters were significantly affected by a small change in SS and were most conspicuous in the SCP and choriocapillaris. These finding highlight the importance of high and consistent SS in quantitative OCTA studies.

## Introduction

Optical coherence tomography angiography (OCTA) is a noninvasive imaging technique that allows visualization of retinal and choroidal blood flow without dye injection^1,2^. The high-contrast and depth resolution of OCTA allows the visualization of three-dimensional microvasculature, including the retinal capillary networks and choriocapillaris (CC). The high contrast also facilitates the quantitative measurement of macular blood flow from the *en face* OCTA images. The quantification of retinal and CC blood flow has provided us with a better understanding of previous angiographic and histologic findings in macular diseases and normal aging^3–5^.

However, because OCTA images can be affected by many factors, such as image quality, motion artifact, projection from overlying vessels, and segmentation error, the analysis of flow should be cautiously interpreted^6^. One commonly used measure of the quality of OCTA images, which reflects the level of noise in the images, is the signal strength (SS). Although different devices can use different scales or ranges, SS is commonly measured from 0 to 10, and a value of 10 indicates the highest signal-to-noise ratio from normal healthy eyes. The SS decreases in the presence of media opacity, such as cataracts, corneal clouding, or vitreous abnormalities. A small pupil, dry eye, and misalignment of the scan can also cause vignetting and decrease in SS^7^. Such a decrease in the SS can impact the vascular images and lead to erroneous quantification of vessel metrics. When the thresholding is applied to generate the flow images from repeated B-scans, low or noisy pixels are removed by the algorithm of OCTA devices^8^. As SS decreases, small flow signals are attenuated, which can be masked with mixing in background noise^9^. This masking can result in flow areas with low signal being displayed as black (no flow). Therefore, many researchers recommend OCTA images with an SS of 8 or better, which is considered as a threshold criterion for selecting reliable images for quantifying blood flow signal^10–14^. However, it is not known to what extent a change in SS can impact the flow metrics of OCTA, even with an SS of 8 or higher, in different devices.

Herein, we artificially changed the SS using commercially available neutral density filters (NDFs) and evaluated the impact of the SS change on the quantitative flow metrics obtained using spectral-domain (SD) and swept-source OCTA (SS-OCTA) devices that were commercially available.

## Results

A total of 20 healthy participants (5 males and 15 females) were enrolled in this study. The mean age was 32.2 ± 6.3 years. The mean spherical equivalent was − 1.08 ± 1.21 diopters.

### Changes in the signal strength and optical coherence tomography angiography images when the neutral density filter was applied

For SD-OCTA, the mean SS incrementally decreased from 10.0 (no filter) to 8.2 (NDF 0.3) (p < 0.001, Wilcoxon signed rank test with post-hoc Bonferroni correction), and from 8.2 (NDF 0.3) to 4.0 (NDF 0.6) (p < 0.001) when NDFs were applied. For SS-OCTA, the mean SS decreased from 10.0 (no filter) to 9.5 (NDF 0.3) (p = 0.005), and from 9.5 (NDF 0.3) to 7.2 (NDF 0.6) (p < 0.001). Table 1 summarizes the results of the quantitative flow metrics in the SCP, DCP, and CC as the SS was reduced using the NDF’s.

**Table 1:** The quantitative measurement of blood flow in the superficial capillary plexus, deep capillary plexus and choriocapillaris flow deficits according to the application of neutral density filter.

	No NDF	NDF 0.3	NDF 0.6	*P*-value	*P*-value	*P*-value	*P*-value
**SD-OCTA**							
Signal strength	10	8.2	4	< 0.001^a^	< 0.001^b^	< 0.001^c^	< 0.001^d^
SCP VD, %	37.5 ± 0.88	35.2 ± 1.44	34.4 ± 1.90	< 0.001^e^	< 0.001^f^	0.393^g^	< 0.001^h^
VLD, %	9.8 ± 0.22	9.2 ± 0.34	9.2 ± 0.48	< 0.001^e^	< 0.001^f^	> 0.999^g^	< 0.001^h^
DCP VD, %	39.3 ± 0.60	38.9 ± 0.83	38.9 ± 1.06	0.205^e^	0.310^f^	> 0.999^g^	0.346^h^
VLD, %	11.2 ± 0.18	11.1 ± 0.21	11.3 ± 0.24	0.011^e^	0.230^f^	0.024^g^	0.481^h^
CCFD, %	30.4 ± 5.95	41.6 ± 5.00	53.4 ± 2.99	< 0.001^e^	< 0.001^f^	< 0.001^g^	< 0.001^h^
**SS-OCTA**							
Signal Strength	10	9.5	7.2	< 0.001^a^	0.005^b^	< 0.001^c^	< 0.001^d^
SCP VD, %	38.1 ± 0.52	37.2 ± 0.99	36.0 ± 1.62	< 0.001^e^	0.001^f^	0.003^g^	< 0.001^h^
VLD, %	11.6 ± 0.24	11.2 ± 0.34	10.8 ± 0.47	< 0.001^e^	0.001^f^	< 0.001^g^	< 0.001^h^
DCP VD, %	36.4 ± 0.87	35.9 ± 0.88	34.5 ± 0.99	< 0.001^e^	0.237^f^	< 0.001^g^	< 0.001^h^
VLD, %	12.6 ± 0.33	12.3 ± 0.33	11.8 ± 0.38	< 0.001^e^	0.006^f^	< 0.001^g^	< 0.001^h^
CCFD, %	22.7 ± 2.09	28.8 ± 2.74	39.2 ± 2.68	< 0.001^e^	< 0.001^f^	< 0.001^g^	< 0.001^h^

*CCFD* choriocapillaris flow deficits, *DCP* deep capillary plexus, *NDF* neutral density filter, *OCTA* optical coherence tomography angiography, *SCP* superficial capillary plexus, *SD* spectral-domain, *SS* swept-source, *VD* vessel density, *VLD* vessel length density.

^a^The p-value was obtained from the Friedman test.

^b^The p-value was obtained from the Wilcoxon signed rank test with post-hoc test (Bonferroni) between no NDF and NDF 0.3 groups.

^c^The p-value was obtained from the Wilcoxon signed rank test with post-hoc test (Bonferroni) between NDF 0.3 and NDF 0.6 groups.

^d^The p-value was obtained from the Wilcoxon signed rank test with post-hoc test (Bonferroni) between no ND and NDF 0.6 groups.

^e^The p-value was obtained from the one-way repeated measures analysis of variance.

^f^The p-value was obtained from the post-hoc test (Bonferroni) between no NDF and NDF 0.3 groups.

^g^The p-value was obtained from the post-hoc test (Bonferroni) between NDF 0.3 and 0.6 groups.

^h^The p-value was obtained from the post-hoc test (Bonferroni) between no NDF and NDF 0.6 groups.

**Figure 1:**
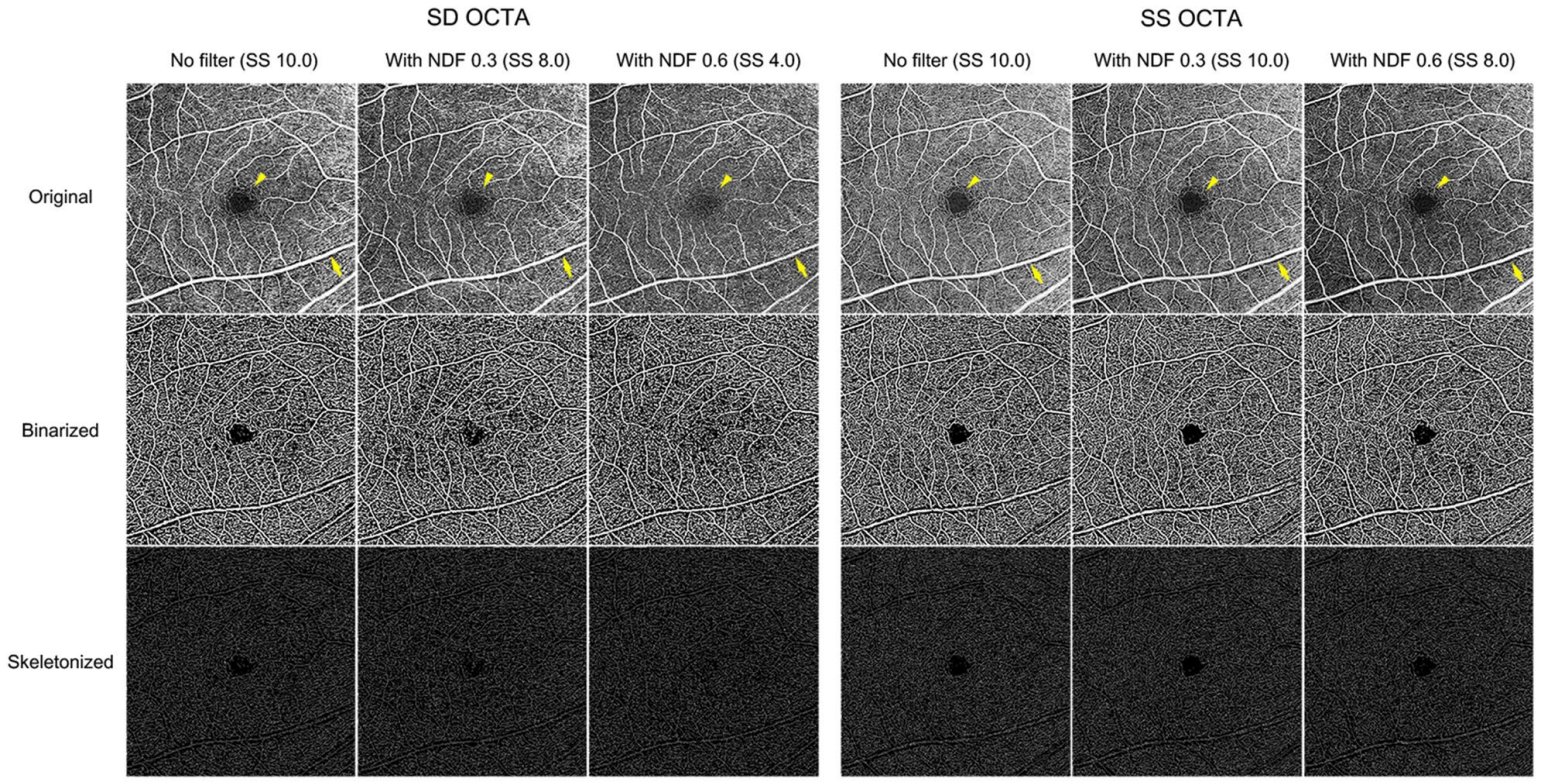
Representative 6 × 6 mm *en face* superficial capillary plexus (SCP) optical coherence tomography angiography (OCTA) images with application of neutral density filter (NDF) of a 35-year-old healthy male subject. Signal strength (SS) decreased significantly with spectral domain OCTA (SD-OCTA) compared to swept-source OCTA (SS-OCTA). The large retinal vessels tended to be narrower (yellow arrow), and the fovea avascular zone appeared fuzzier (yellow arrowhead) when SS decreased. Such SCP image changes were more obvious in the SD-OCTA than in the SS-OCTA. The vessel density (VD) was 38.0%, 36.1%, and 35.7%, and the vessel length density (VLD) was 9.9%, 9.3%, and 9.4% when SS changed, respectively. For SS-OCTA, the VD was 38.0%, 36.7%, and 36.2%, and the VLD was 11.5%, 11.1%, and 10.8%, respectively.

**Figure 2:**
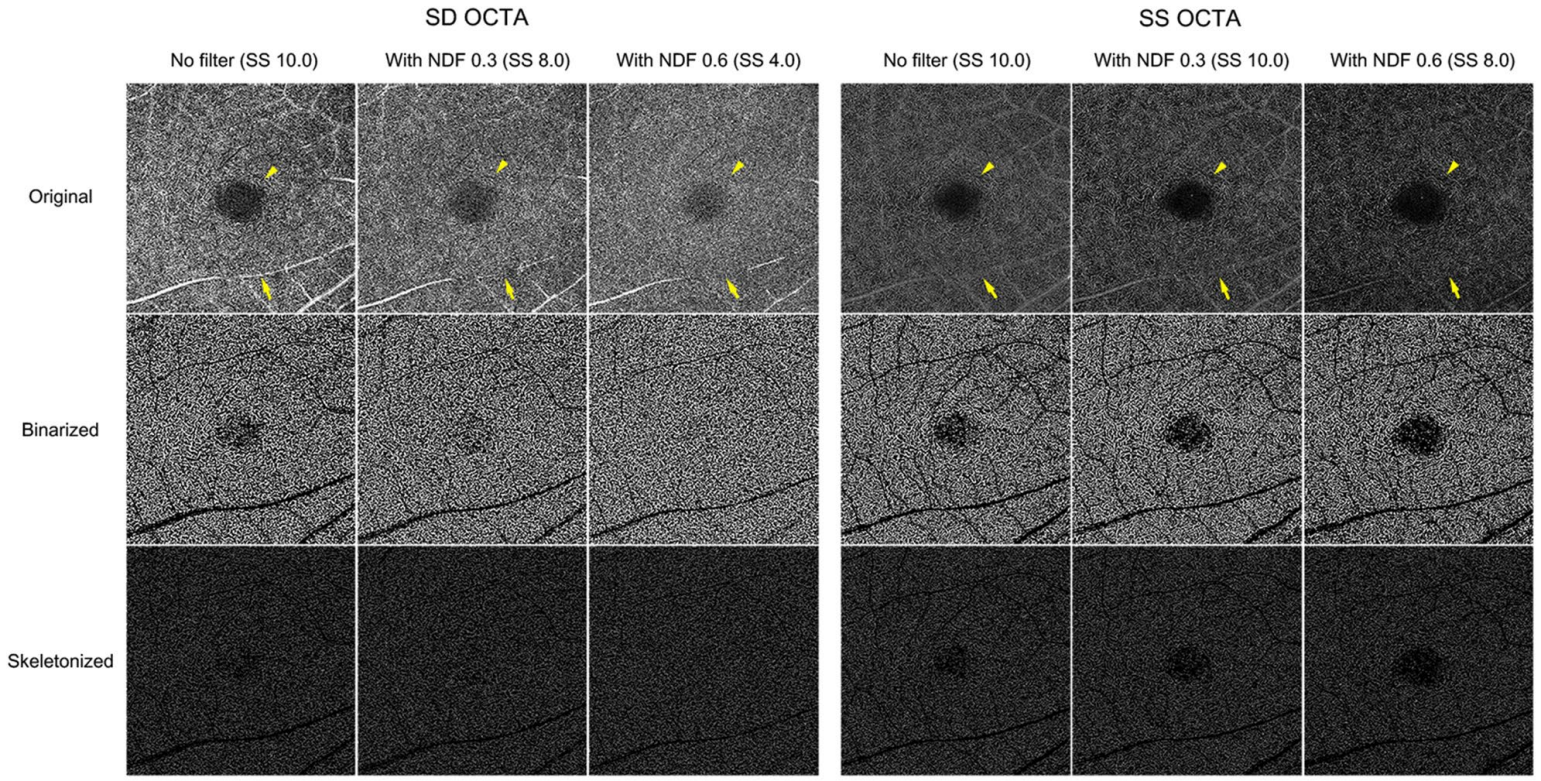
Representative 6 × 6 mm *en face* deep capillary plexus (DCP) optical coherence tomography angiography (OCTA) images with application of neutral density filter (NDF) of a 35-year-old healthy male subject. Signal strength (SS) decreased significantly for spectral domain OCTA (SD-OCTA) compared to swept-source OCTA (SS-OCTA). The boundaries of the fovea avascular zone became unclear (yellow arrowhead), and projection of overlying retinal vessels was faint (yellow arrow) when SS decreased. These features were more prominent for SD-OCTA than for SS-OCTA. For SD-OCTA, the vessel density (VD) was 39.3%, 37.8%, and 37.8%, and the vessel length density (VLD) was 11.1%, 10.8%, and 11.0% when SS decreased, respectively. For SS-OCTA, the VD was 33.7%, 34.1%, and 31.6%, and the VLD was 12.5%, 12.3%, and 11.4%, respectively.

**Figure 3:**
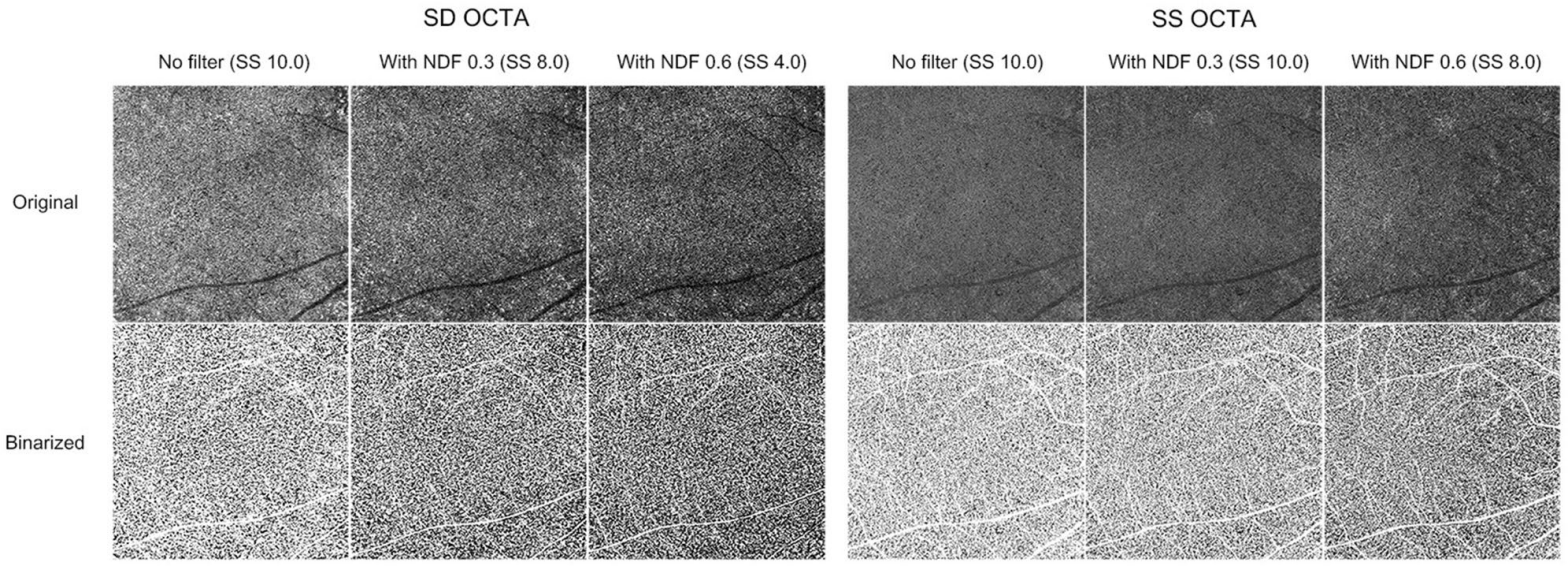
Representative 6 × 6 mm *en face* choriocapillaris (CC) optical coherence tomography angiography (OCTA) images with application of neutral density filter (NDF) of a 35-year-old healthy male subject. Signal strength (SS) decreased significantly more for spectral domain OCTA (SD-OCTA) than swept-source OCTA (SS-OCTA). The CC images became dark since the areas of black pixels increased when SS decreased. The CC flow deficit (CCFD) increased from 31.4–42.0 to 53.7% for SD-OCTA, and from 22.4–28.3 to 38.7% for SS-OCTA when SS decreased, respectively.

The appearance of the vessels on the OCTA images differed between SD- and SS-OCTA devices, even with the same magnitude of SS decrease. For SD-OCTA, the large retinal vessels tended to be narrower, the small vessels faded and disappeared in the SCP and DCP, and the fovea avascular zone (FAZ) was blurred and brighter as the SS decreased. In contrast, for SS-OCTA images, though the images were darker, the retinal vasculature was relatively clear and the boundary of the FAZ remained distinct, albeit with a slight increase in area (*en face* OCTA images of SS 8.0 in Figs. 1, 2). The projection of overlying large retinal vessels decreased in the DCP (Fig. 2). The CC images obtained from both devices showed similar changes. When the SS decreased, the image pixels of capillary components became sparse, and signal void areas presenting inter-capillary spaces enlarged. The overall CC features became darker and less distinct. The shadow from the overlying retinal vessels increased (Fig. 3).

### Changes in the quantitative flow parameters of the superficial capillary plexus

For SD-OCTA, the mean VD of the SCP decreased from 37.5 to 35.2% (p < 0.001, one-way repeated measures analysis of variance (RMANOVA) with post-hoc Bonferroni correction) when the SS changed from 10.0 to 8.2. However, there was no further significant decrease in VD (34.4% with SS of 4.0) when SS decreased further from 8.2 to 4.0. The mean VLD of the SCP significantly decreased from 9.8 to 9.2% (p < 0.001) when the SS decreased from 10.0 to 8.2. However, there was no further decrease in VLD (9.2% with SS of 4.0) when SS decreased further from 8.2 to 4.0. For SS-OCTA, the mean VDs (38.1%, 37.2%, and 36.0%) and VLDs (11.6%, 11.2%, and 10.8%) of the SCP incrementally decreased in parallel with the decrease in SS (p < 0.05 in all). The representative images of the SCP obtained when NDFs were applied are shown in Fig. 1.

### Changes in quantitative flow parameters of the deep capillary plexus

For SD-OCTA, the mean VDs (39.3%, 38.9%, and 38.9%, respectively) of the DCP did not change as the SS was reduced. The mean VLDs were 11.2%, 11.1%, and 11.3% as the SS decreased, respectively.

For SS-OCTA, the mean VDs (36.4%, 35.9%, and 34.5%) and VLDs (12.6%, 12.3%, and 11.8%) of the DCP decreased in parallel with the decrease in SS (p < 0.05 in all, one-way RMANOVA with post-hoc Bonferroni correction), with the exception of changes in VD (36.4% to 35.9%) when the SS changed from 10 to 9.5. The representative images of the DCP when NDFs were applied are shown in Fig. 2.

### Changes in signal strength and choriocapillaris flow deficit

For SD-OCTA, the mean CCFDs (30.4%, 41.6%, and 53.4%) significantly increased as the SS decreased (p < 0.001 in all, one-way RMANOVA with post-hoc Bonferroni correction, respectively). For SS-OCTA, the mean CCFDs (22.7%, 28.8%, and 39.2%) also increased significantly (p < 0.001 in all). The representative images of the CC when NDFs were applied are presented in Fig. 3.

### Correlation between signal strength and quantitative flow measurements

The SS change was significantly correlated with the changes in flow parameters obtained with both OCTA devices, except for the DCP with the SD-OCTA. Table 2 shows the correlations between changes in SS and flow parameters.

**Table 2:** The correlations between signal strength and quantitative flow parameters.

	SD-OCTA (Cirrus)		SS-OCTA (PlexElite)	
	R	*P*-value	R	*P-*value
SCP VD	0.408	< 0.001^a^	0.488	< 0.001^a^
VLD	0.316	< 0.001^a^	0.578	< 0.001^a^
DCP VD	0.080	0.286	0.688	< 0.001^a^
VLD	− 0.144	0.054	0.677	< 0.001^a^
CCFD	− 0.857	< 0.001^a^	− 0.898	< 0.001^a^

*CCFD* choriocapillaris flow deficits, *DCP* deep capillary plexus, *OCTA* optical coherence tomography angiography, *SCP* superficial capillary plexus, *SD* spectral-domain, *SS* swept-source, *VD* vessel density, *VLD* vessel length density.

^a^P < 0.05 was considered statistically significant. R is Pearson correlation coefficient.

## Discussion

**Figure 4:**
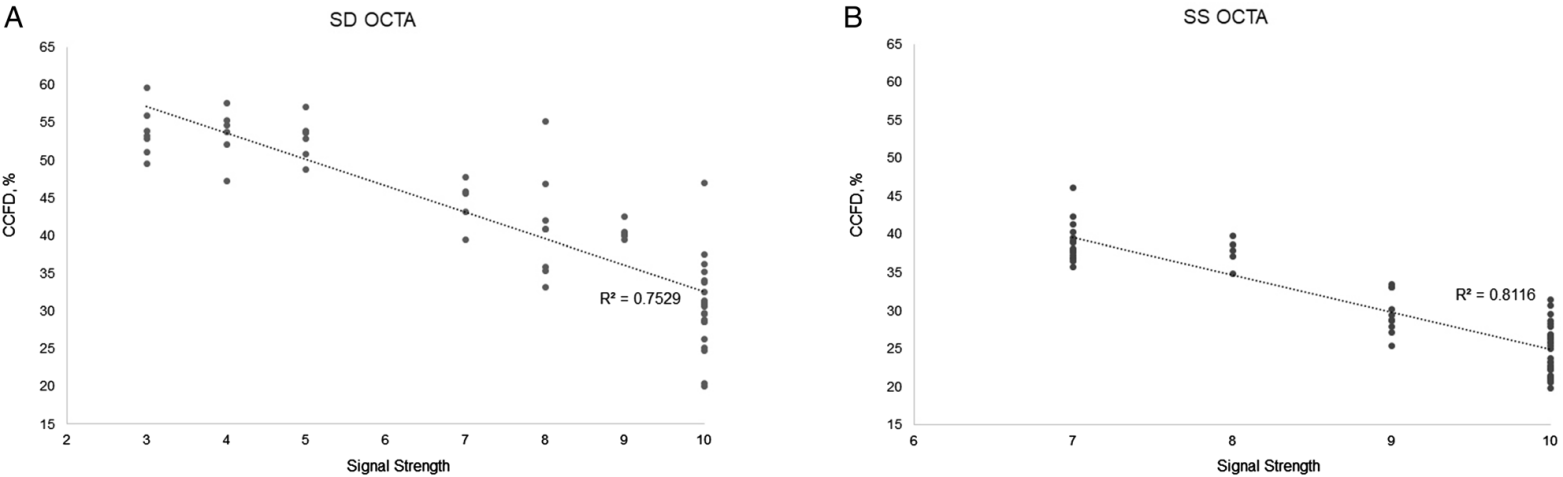
Scatter plots of the association between signal strength and choriocapillaris flow deficits (CCFD) ((**A**) spectral domain optical coherence tomography (SD-OCTA), (**B**) swept-source OCTA). The CCFD decreased in parallel with the SS decrease following a linear trend on both devices.

This study demonstrated that SS was a significant factor influencing the quantitative flow signal metrics of OCTA, even when the SS remained within the range of a high or acceptable quality OCTA image (i.e. SS > 8). The SS decreased when the NDF was applied, and consequently the qualitative appearance of the *en face* OCTA images changed. The retinal vessels became faint, and the boundary of FAZ became fuzzy and indistinct. The projection of overlying retinal vessels faded. The CC images became darker owing to the increase of black pixels. This was more apparent with the SD-OCTA than with the SS-OCTA. Regarding quantitative flow metrics, the flow parameters of the SCP were significantly affected and decreased. The CCFD decreased in parallel with the SS decrease following a linear trend (Fig. 4). As noted, an SS of 8 or higher could still be associated with a significant change in flow parameters, compared to an SS of 10. However, SS change did not affect the flow metrics of the DCP as much as those of the SCP and CC, especially for the SD-OCTA device, although the qualitative appearance of the DCP OCTA images did appear to change. Changes in the SS were significantly correlated with changes in the flow parameters in the SCP and CC for both OCTA devices.

OCTA enables visualization of flow in the retinal and choroidal vasculature by detecting the motion contrast between repeated B-scans^2,15,16^. It also the flow in specific vascular layers of interest to be visualized, such as the SCP, DCP, and CC. We used the devices’ default settings for each slab in this study. Such flow images (*en face* OCTA images in this study) can be used to quantify the VD, VLD, and CCFD using simple binarization techniques, in which pixels of an image are divided into pixels with or without flow.

The SS, which impacts image quality, is a significant factor in the quantification of the retinal and CC blood flow obtained from OCTA, since actual vessel metrics depend on the image quality. Most OCTA studies have deemed an SS of 8 or better as sufficient for quantitatively analyzing flow^10–14^. However, signals of 8 or better generated different appearing images and significantly different vessel metrics from the same eye in the current study. There are several studies on SS change and its effect on OCTA analysis. Lim et al.^7^ divided 446 healthy eyes into four groups, based on a signal intensity of 7, 8, 9, and 10, and compared the VDs of the SCP. The VDs significantly differed among the four groups. Based on this finding, they recommended an SS of at least 9 to be used for accurate quantitative analysis. However, true variation in flow between individuals could not be determined in that study, it was difficult to precisely evaluate the relationship between SS and the vessel metrics. To address this issue, Al-Sheikh et al.^17^ used topical ointment to reduce the SS artificially. In cases of ointment application, both SS and OCTA image quality decreased. They observed that the VD decreased in the SCP and increased in the DCP. However, topical ointment may not provide a consistent decrease in SS. Their vasculature images generated by topical ointment appeared to be smeared because of the scattering of the laser beam. To generate a reliable and consistent degree of reduction in the SS, we used a NDF that could filter 700–1100 nm wavelength lights. NDFs 0.3 and 0.6 were designed to filter 50% and 75% of light, respectively. Therefore, the NDFs could consistently decrease the SS of both SD- (wavelength: 840 nm) and SS-OCTA (wavelength: 1040–1080 nm) in the present study. Gao et al.^18^ previously applied the NDF to artificially change the reflectance of SD-OCTA images in five healthy participants. They found that the NDF lowered the SS and image reflectance and consequently decreased the VD of SCP. In the current study, we additionally found that the SS decrease was greater in SD-OCTA than in SS-OCTA. The signal of the SS-OCTA device was less affected by the NDF, which could be derived from a greater number of A-scan compared to SD-OCTA. An interesting finding in our study was that SD- and SS-OCTA devices yielded different changes in the OCTA image with the same decrease in SS. When the SS decreased to 8.0 for both devices (NDF 0.3 in SD-OCTA and NDF 0.6 in SS-OCTA, respectively), the retinal vessels tended to be narrower, and the FAZ appeared fuzzier in the SD-OCTA, whereas the retinal vasculature was relatively clear and the boundary of the FAZ was distinct in the SS-OCTA (Figs. 1, 2). The CC became sparser with enlarged inter-capillary spaces in the SD-OCTA than in the SS-OCTA (Fig. 3). The projection from the overlying retinal vessels faded in the DCP, and the shadows of overlying retinal vessels became more pronounced in the CC using both SD- and SS-OCTA devices (Figs. 2, 3). Possible explanations for this discrepancy are as follows. The SD-OCTA device (Cirrus HD-OCT 5000) operates at 68,000 A-scans per second, an A-scan depth of 2.0 mm, and axial resolution of 5 µm, and a transverse resolution of 15 µm^19^. For the 6 × 6 mm SD-OCTA scans, each B-scan contained 350 A-scans per B-scan along the fast x-axis, and each B-scan was repeated two times at each position. There were 350 B-scan positions along the slow y-axis. As a result, *en face* SD-OCTA images have a homogenous sampling grid with a separation of 17.1 µm^19^. The SS-OCTA device (PLEX Elite 9000) operates at 100,000 A-scans per second, an A-scan depth of 3.0 mm, and axial resolution of 6.3 µm, and a transverse resolution of 20 µm^19^. This device allows for faster scanning speed and reduced sensitivity loss with deeper layer imaging that more detailed scan of all retinal layers can be possible^20,21^. The 6 × 6 mm SS-OCTA scans use 420 A-scans per B-scan repeated twice at each of the 420 B-scan locations. As a result, SS-OCTA images have a homogenous sampling grid with a separation of 14.3 µm^19^. Regarding these features, the difference in the devices’ technology could contribute to the difference in the OCTA images with the same SS decrease.

The qualitative changes in OCTA images accompanying the SS decrease influenced the quantitative flow measurements. In the present study, different flow values were computed from the OCTA images with SS 8 or higher, compared to those with SS 10. The VD and VLD in the SCP significantly decreased with the small SS decrease using both SD- and SS-OCTA. However, no further changes were observed when the SS changed from 8.2 to 4.0, although the qualitative vascular features of the SD-OCTA images were very different. The flow parameters in the DCP were less affected by SS change. A possible explanation for this discrepancy is that large retinal vessels became narrow, and small retinal capillaries were attenuated and disappeared when the SS decreased. The decreased flow signals turned to black pixels when binarization was applied to these lower quality OCTA images. Therefore, the flow area in the SCP containing large retinal vessels was significantly reduced by a small decrease in the SS, and vessel metrics also decreased. However, the FAZ became fuzzier in the OCTA image and brighter in the binarized one when the SS decreased further [binarized image of NDF 0.6 (SS 4.0) in Figs. 1, 3]. This brightness change in the FAZ was miscalculated as the flow area and could compensate for the reduction of the vessel area when the SS decreased further. This scenario seemed to occur in the DCP where no decrease in the flow parameters was observed despite a decrease in SS. The CC structure is quite different from the retinal vasculature. The CC is a capillary meshwork with tiny inter-capillary spaces and rough hexagonal-shaped lobules. The lobules at the posterior pole are smaller compared to those at the periphery of the eye. The OCTA CC image is composed of alternating black and white pixels similar to that of a fine ground glass appearance. In the current study, the capillary component appeared to attenuate, and the inter-capillary space increased as the SS decreased. The CC image overall became dark. Such changes were similar to those observed with aging^22^, wherein an increased proportion of black pixels and high CCFD% in the binarized images are observed.

Our study has a number of limitations to be considered when assessing our results. First, our sample was relatively small—on the other hand, despite this we still observed significant changes in vessel parameters, further highlighting the importance of differences in SS. Furthermore, we only modulated SS whereas there a number of other artifacts including local attenuation artifact, projection artifact, and motion artifacts that may also impact OCTA image quality^9^. To control factors associated with image artifacts, we used several techniques in this study. To reduce the projection artifact from overlying vasculature, the devices’ projection removal function and masking the area of superficial large vessels were applied. Each subject was scanned three times, and the vessel metrics were averaged to minimize differences between scan caused by dynamic variations in blood flow. To reduce motion artifact, FastTrac motion correction that was embedded in the device was applied. We also used SD-OCTA and SS-OCTA devices and their default settings for slab selection. The type of device and different slab positions could produce different results. Another limitation is that while we used NDF to simulate the impact of media opacity, NDF’s cause a uniform reduction in signal across the scan field whereas naturally occurring media opacities such as cataract may have an inhomogeneous impact with varying reductions in signal in different regions of the scan area. Finally, the binarization method including local or global thresholding and brightness/contrast adjustment might also affect the quantitative flow measurements^23,24^, and different thresholding approaches may behave differently in the face of changes in SS. On the other hand, there is still no universally accepted thresholding standard, and the approach we used is one that is commonly described in the literature^24–27^.

In summary, we observed that the quantitative OCTA flow parameters were significantly affected by a small change in SS, even with an overall high signal, especially in the SCP and CC. The impact of SS changes on the flow parameters was different between SD- and SS-OCTA devices. Our results highlight the importance of maintaining not only high, but also a consistent SS. This may be of particular importance when comparing OCTA vessel metrics over time.

## Methods

This prospective, cross-sectional, comparative study was conducted at the Pusan National University Hospital between January 2021 and June 2021. OCTA images were obtained from healthy volunteers. This study was approved by the Institutional Review Board of Pusan National University Hospital and was conducted in accordance with the principles outlined in the tenets of the Declaration of Helsinki. Written informed consent was obtained from all subjects prior to enrollment in the study. All participants underwent a comprehensive ophthalmic examination, including best corrected visual acuity measurement, intraocular pressure, slit-lamp examination, autorefractor keratometer, SD-OCTA, and SS-OCTA. Healthy volunteers without any ocular diseases were enrolled in this study. Subjects with media opacity, refractive error greater than ± 2 diopters, and astigmatism greater than 1.0 diopter were excluded. Subjects with systemic diseases such as diabetes mellitus, hypertension, and dyslipidemia were also excluded.

### Imaging and modulation of signal strength

Subjects were imaged using both commercially available SD-OCTA (Cirrus HD-OCT 5000, Carl Zeiss Meditec, Inc., Dublin, CA, USA) and SS-OCTA (PLEX Elite 9000, Carl Zeiss Meditec, Inc., Dublin, CA, USA) devices. The right eye of each subject was scanned three times using a 6 × 6 mm volume scan protocol with FastTrac motion correction centered on the fovea in each OCTA device. Next, the near-infrared neutral density filter (NDF, Edmund Optics, Barrington, NJ, USA) was positioned on the OCTA device, and subjects were scanned three separate times using the same scan protocol. NDFs 0.3 and 0.6, designed to reduce the illumination light of 700-1100 nm wavelength by 50% and 75%, respectively, were applied. Topical lubricants were used to optimize ocular surface stability before every scan.

### Quantitative flow assessment of the superficial capillary plexus, deep capillary plexus, and choriocapillaris in the *en face* optical coherence tomography angiography images

The *en face* OCTA images of the superficial capillary plexus (SCP), deep capillary plexus (DCP), and CC were generated using the device’s default setting. The CC slab was extracted following semi-automatic segmentation of the retinal pigment epithelium/Bruch’s membrane complex. In the DCP and CC, the projection from overlying vessels was removed using the embedded function of the devices.

**Figure 5:**
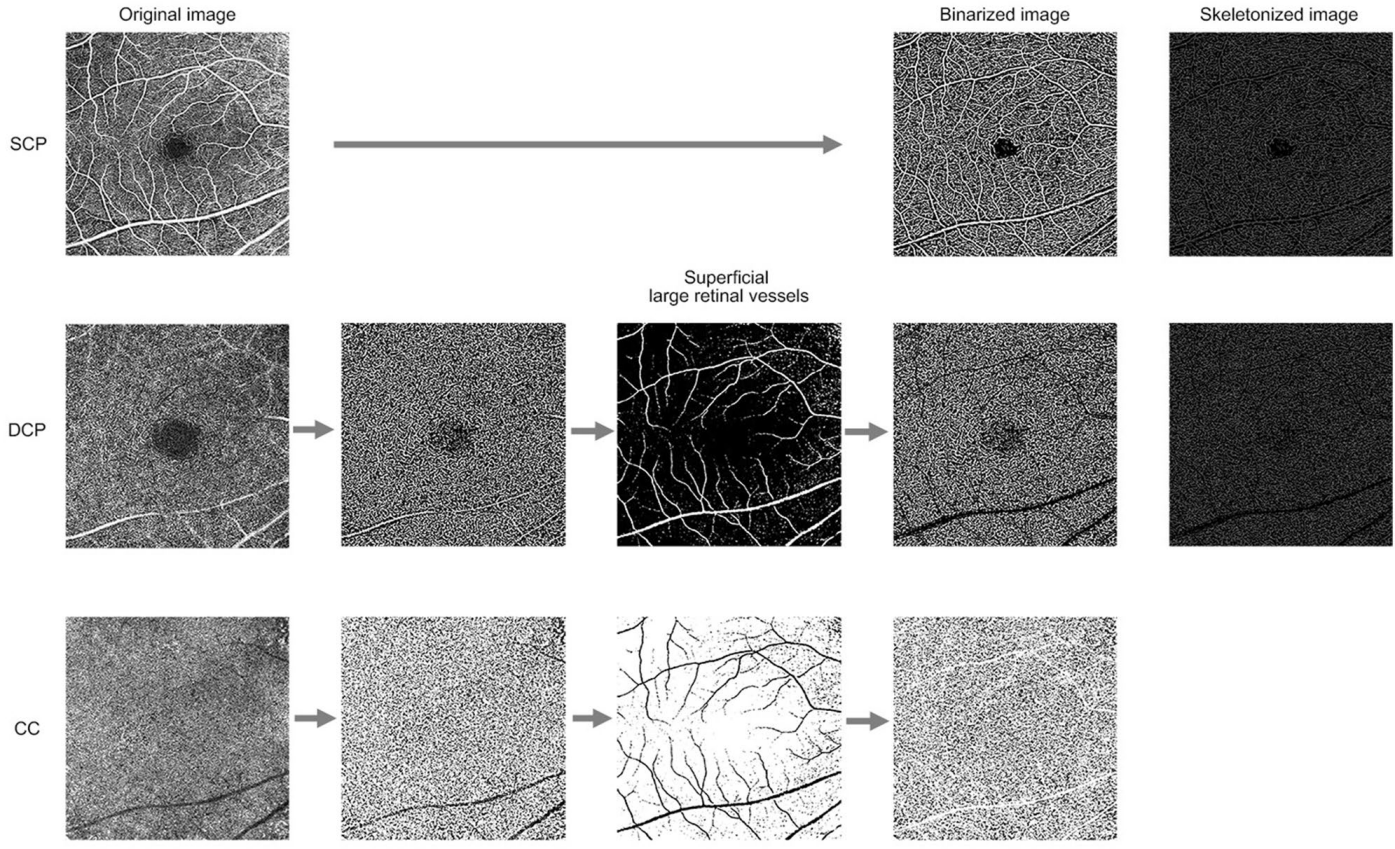
Representative flow chart of the optical coherence tomography angiography image processing of the superficial capillary plexus (SCP), deep capillary plexus (DCP), and choriocapillaris (CC) using FIJI software (an expanded version of ImageJ), version 1.52u (National Institutes of Health, Bethesda, MD, USA). The SCP and DCP images were duplicated, and one image was binarized with two different threshold values (“hessian” and “Huang’s fuzzy”, respectively). The other image was binarized using median local thresholding. The resultant binarized image was then generated, and only pixels that existed on two binarized images were included. The MaxEntropy threshold was applied to binarized SCP images. The obtained thresholded images were then merged with binarized DCP and CC images for identification and removal of the superficial retinal large vessels.

The *en face* OCTA images of all layers were binarized using FIJI software (an expanded version of ImageJ), version 1.52u (National Institutes of Health, Bethesda, MD, USA; available at http://rsb.info.nih.gov/ij/index.html), as previously described^5^. In brief, both the SCP and DCP images were duplicated. One image was binarized with two different thresholds (“hessian” and “Huang’s fuzzy”, respectively). The other image was binarized using median local thresholding. The two different threshold images were combined to generate the final binarized image in which only those pixels existing on both of the images were included^5,28^. To avoid the confounding effect from projection artifact by superficial large retinal vessels, the MaxEntropy threshold was applied to binarized SCP images. The obtained thresholded images were then merged with binarized DCP and CC images using FIJI software for identification and removal of the superficial retinal large vessels^5^. Vessel density (VD) and vessel length density (VLD) were measured in the SCP and DCP and flow deficits were computed from the CC as previously described^5,10,13,28,29^. VD was defined as the percentage area occupied by blood vessels and was assessed on the final image obtained. After binarization, images were skeletonized in order to obtain an image in which vessels were visualized as a trace of 1 pixel in width. The obtained images were used for quantitative analysis of the VLD, which represents the vessel length per unit area^5,13,28,29^. The *en face* CC OCTA image was composed of alternating white and black pixels. For each pixel, if its value was higher than its threshold value, the pixel would appear as white on the binarized image, indicating the presence of blood flow. The CC flow deficits (CCFD) shown as black pixels in the binarized images was computed as a percentage of the total area^10^. The *en face* CC OCTA images were segmented using Phansalkar’s local thresholding method, with a local window radius of 15 pixels (43.94 µm). The resultant *en face* OCTA images are shown in Fig. 5.

### Statistical analysis

All statistical analyses were performed using IBM SPSS Statistics for Windows, Version 23.0 (IBM Corp., Armonk, NY, USA). The mean value of three repeated scans was used for statistical analysis. The Kolmogorov–Smirnov and Shapiro–Wilk tests were used for normality testing. After the normality test, the Friedman and the Wilcoxon signed rank tests with post-hoc Bonferroni correction were used for non-normal distributions, and one-way RMANOVA with post-hoc Bonferroni correction was performed for normal distributions. The SS values for no filter, NDF 0.3, and NDF 0.6 that exhibited non-normal distribution were compared using the Friedman test and the Wilcoxon signed rank tests with post-hoc Bonferroni correction. To compare the quantitative flow parameters in the SCP, DCP, and CC that presented a normal distribution, one-way RMANOVA with post-hoc Bonferroni correction was performed. Pearson correlation and linear regression were used to test the association between SS and the quantitative flow parameters. A p-value < 0.05 was considered statistically significant.

## Data Availability

Data supporting the findings of the current study are available from the corresponding author on reasonable request.
